# An Essay on the Influence of Air and Soil as Affecting Health

**Published:** 1837-04

**Authors:** 


Art. XVIII.—*An Essay on the Influence of Air and Soil as affecting Health; to which was adjudged the Prize offered by the* Reverend Thomas Arnold, d.d., *to the Students of the Royal School of Medicine and Surgery at Birmingham.* By Alexander Wright, Student.—*Birmingham*, 1836. 8vo. pp. 56.

This little essay is creditable alike to its youthful author, the school whence it originated, and the distinguished scholar whose liberality is the cause of its being written. It contains a concise, well-written, and well-arranged digest of our knowledge on the subjects of which it treats, but affords nothing new for quotation. More than once we have taken upon us to condemn the over-anxiety of young writers to come before the public; and we can only excuse the present publication on the score of the novelty of the occasion, and the particular circumstances attending it. The honour of having written an essay that would do no discredit to his seniors, may not only prove a valuable stimulus to Mr. Wright to exert himself through life, but may excite others to follow his example, and thus benefit his fellows and his school. It is most gratifying to us to observe the progressive advance of the provincial schools, in the successful prosecution of medical education; and we look upon the essay of Mr. Wright as a very favorable sign of the times.

